# Second breast cancer: recurrence score results, clinicopathologic characteristics, adjuvant treatments, and outcomes—exploratory analysis of the Clalit registry

**DOI:** 10.1038/s41523-023-00586-3

**Published:** 2023-09-30

**Authors:** Shlomit S. Shachar, Michelle Leviov, Rinat Yerushalmi, Karen Drumea, Margarita Tokar, Lior Soussan-Gutman, Avital Bareket-Samish, Amir Sonnenblick, Noa Ben-Baruch, Ella Evron, Einav Nili Gal-Yam, Shani Paluch-Shimon, Gil Bar-Sela, Hadar Goldvaser, Salomon M. Stemmer

**Affiliations:** 1 Sourasky Medical Center, Tel Aviv, Israel; 2 Tel Aviv University, Tel Aviv, Israel; 3 Lin Medical Center, Haifa, Israel; 4 Rabin Medical Center, Petah Tikva, Israel; 5 Soroka University Medical Center and Ben Gurion University, Beer Sheva, Israel; 6 Oncotest-Rhenium, Modi’in, Israel; 7 BioInsight Ltd, Binyamina, Israel; 8 Kaplan Medical Center, Rehovot, Israel; 9 Hebrew University Medical School, Jerusalem, Israel; 10 Sheba Medical Center, Ramat Gan, Israel; 11 Hadassah Hebrew University Medical Center, Jerusalem, Israel; 12 Emek Medical Center, Afula, Israel; 13 Rappaport Faculty of Medicine, Technion, Haifa, Israel; 14 Shaare Zedek Medical Center, Jerusalem, Israel

**Keywords:** Breast cancer, Cancer therapy

## Abstract

Data on using the 21-gene Recurrence Score (RS) testing on second breast cancer (BC; second primary or local recurrence) are lacking. This cohort study examined patients with first and second BC, who underwent 21-gene testing both times. It included a ‘study-cohort’ (60 N0/N1mi/N1 ER + HER2‒ BC patients with ≥2 RS results >1 year apart) and a ‘general 21-gene-tested BC-cohort’ (2044 previously described N0/N1mi/N1 patients). The median time between the first and second BC was 5.2 (IQR, 3.1–7.1) years; the second BC was ipsilateral in 68%. Patient/tumor characteristics of the first- and second-BC in the ‘study-cohort’ were similar, except for the RS which was higher in the second BC (median [IQR]: 23 [17–30] vs 17 [14–22], *p* < 0.001). Overall, 56 patients had follow-up data, of whom 5 experienced distant recurrence (2 RS 11–25 patients and 3 RS 26–100 patients). Studies exploring the prognostic utility of the RS in this setting are warranted.

## Introduction

The 21-gene Oncotype DX Breast Recurrence Score^®^ assay is a prospectively-validated tool for guiding adjuvant chemotherapy (CT) decisions in patients with hormone receptor (HR)+ human epidermal growth factor receptor 2 negative (HER2‒) early-stage breast cancer (BC)^1–8^.

In both phase 3 prospective validation studies for the 21-gene assay, a considerable proportion of events involved second primary cancers and local/regional recurrences. In TAILORx, which included 9719 eligible patients with node-negative HR + HER2‒ early-stage BC, patients with Recurrence Score^®^ (RS) results 11–25 were randomized to CT plus endocrine therapy (ET) vs ET alone, whereas all patients with RS 0–10 results received ET alone and all patients with RS 26–100 results received CT plus ET^2^. A total of 1210 events were reported during the study (across all arms), including 413 non-breast second malignancies, 130 contralateral BC, 107 locoregional BC recurrences, and 90 ipsilateral BC^2^. Similarly, in the RxPONDER study which included 4984 eligible patients with node-positive HR + HER2‒ early-stage BC and RS results of 0–25 who were randomized to CT plus ET vs ET alone, 481 events were reported (across treatment arms) including 116 non-breast second malignancies, 56 local/regional recurrences, and 35 contralateral BC^6^.

The Clalit Health Services (CHS) registry includes all RS data for patients tested through CHS (the largest health maintenance organization in Israel) since 2006. Various analyses of the registry data have been published thus far, including 5-year and 10-year outcome data overall and by ethnicity^9–12^.

There are currently no data on using 21-gene testing on second BC (i.e., second primary, local recurrence) and thus no guidelines about its validity and clinical utility in this setting. Therefore, the objectives of the current exploratory analysis of the CHS registry were to investigate the characteristics of patients with second BC vs patients with first BC in the CHS registry, characterize potential differences in clinicopathologic characteristics and RS results between the first and second BC, and investigate clinical outcomes in patients after the second BC.

## Results

### The study cohort vs the general RS-tested BC cohort: patient/tumor characteristics

Between 1/2006 and 12/2020, 11,040 21-gene assays were ordered through CHS for 10,659 unique BC patients. The final study cohort included 60 N0/N1mi/N1 estrogen receptor (ER) + HER2‒ BC patients for whom two 21-gene assays were performed more than a year apart. All patients in the study cohort were female. The median (interquartile range [IQR]) time between the first and second RS result was 5.2 (3.1–7.1) years. In 41 cases (68.3%), the second BC was ipsilateral, and in 19 (31.7%), it was contralateral.

The general 21-gene-tested BC cohort included 1340 N0 and 704 N1mi/N1 female patients (tested between 2006 and 2009 [N0] or between 2006 and 2011 [N1mi/N1]) who were described in prior reports and for which treatments received and clinical outcomes were available^9,11^. Baseline patient/tumor characteristics at the *first* RS testing of the study cohort by nodal status are presented alongside the corresponding characteristics in the general 21-gene-tested BC cohort (Table 1). Overall, the two cohorts were similar, except for age at testing among N0 patients which was statistically significantly younger in the study cohort compared to the general 21-gene-tested BC cohort (median [IQR], 55.5 [43–61] vs 60 [53–66] years, *p* = 0.002) (Table 1).

**Table 1:** Patient and tumor characteristics at the time of first 21-gene testing in the study cohort and the general 21-gene-tested BC cohort by nodal status.

	N0			N1mi/N1		
Characteristics	Study cohort *n* = 46	General 21-gene-tested BC cohort *n* = 1340	p*	Study cohort *n* = 14	General 21-gene-tested BC cohort *n* = 704	p*
Median (IQR) age at testing, years	55.5 (43.3–61.0)	60.0 (52.8–66.0)	**0.002**	55.5 (47.8–65.3)	62.0 (53.0–67.0)	0.15
Median (IQR) tumor size, cm	1.5 (1.0–2.15)	1.5 (1.1–2.0)	0.70	2.0 (1.3–2.4)	1.7 (1.3–2.3)	0.39
Tumor size category, *n* (%)						
≤1 cm	14 (30.4%)	290 (21.6%)	0.33	1 (7.1%)	114 (16.2%)	0.62
>1–2 cm	20 (43.5%)	744 (55.5%)		7 (50.0%)	377 (53.6%)	
>2 cm	12 (26.1%)	296 (22.1%)		6 (42.9%)	205 (29.1%)	
Not available	0 (0)	10 (0.7%)		0 (0)	8 (1.1%)	
Tumor grade category, *n* (%)						
Grade 1	7 (15.2%)	192 (14.3%)	0.44	2 (14.3%)	101 (14.3%)	0.51
Grade 2	28 (60.9%)	674 (50.3%)		4 (28.6%)	380 (54.0%)	
Grade 3	5 (10.9%)	217 (16.2%)		5 (35.7%)	111 (15.8%)	
Not available	6 (13.0%)	257 (19.2%)		3 (21.4%)	112 (15.9%)	
Histology, *n* (%)						
IDC	39 (84.8%)	1082 (80.7%)	0.73	11 (78.6%)	591 (83.9%)	0.67
ILC	3 (6.5%)	159 (11.9%)		2 (14.3%)	85 (12.1%)	
Mucinous/ colloid/papillary	2 (4.3%)	54 (4.0%)		0 (0)	11 (1.6%)	
Not available	2 (4.3%)	45 (3.4%)		1 (7.1%)	17 (2.4%)	
Nodal status, *n* (%)						
N0	46 (100%)	1340 (100%)	NA	0 (0)	0 (0)	
N1mi	0 (0)	0 (0)		6 (42.9%)	294 (41.8%)	0.71
N1	0 (0)	0 (0)		8 (57.1%)	410 (58.2%)	

*IDC* invasive ductal carcinoma, *ILC* invasive lobular carcinoma, *IQR* interquartile range, *NA* not applicable.

*p value for Mann-Witney and chi-square test for continuous and categorical variables, respectively. Bolded *p* values are statistically significant.

### The study cohort vs the general 21-gene-tested BC cohort: RS results

**Fig. 1:**
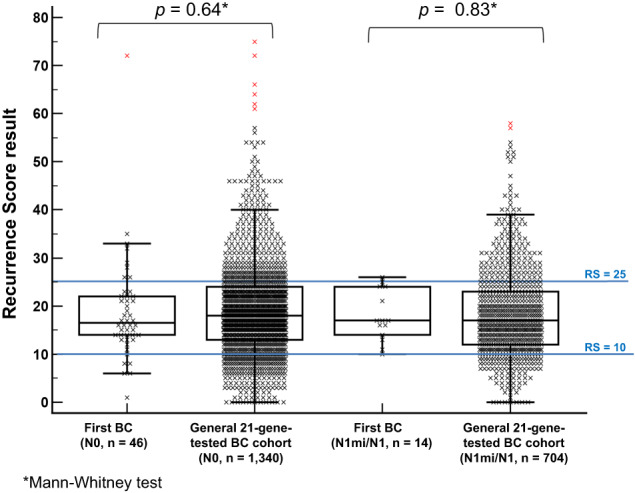
A Box-and-Whiskers plot showing the RS distribution in the first BC in the study cohort vs the RS distribution in the ‘general 21-tested BC cohort’ by nodal status. The horizontal line represents the median, and the box is drawn from the 1^st^ to the 3^rd^ quartile. The upper whisker represents the highest value below the 3^rd^ quartile plus 1.5 × IQR. The lower whisker represents the lowest value above the 1^st^ quartile minus 1.5 × IQR. Red x symbols represent datapoints that are beyond the 3^rd^ quartile plus 3 × IQR. BC breast cancer.

The distribution of the RS results in the first BC diagnosis in the study cohort vs those in the general 21-gene-tested BC cohort by nodal status is presented in Fig. 1. No statistically significant differences in the median RS result were observed: the median (IQR) RS was 17 (14–22) in the study cohort vs 17 (14–22) in the 2044 general 21-gene-tested BC cohort (*p* = 0.80). Among the N0 patients, the median (IQR) RS result was 16.5 (14–22) in the study cohort and 19 (13–24) in the general 21-gene-tested BC cohort (*p* = 0.64), whereas, among the N1mi/N1 patients, the median (IQR) RS results were 17 (15–23) and 17 (12–23), respectively (*p* = 0.83). RS distribution based on the TAILORx^1^ categories also did not differ significantly between the study cohort and the corresponding populations in the general 21-gene-tested BC cohort. In the study cohort, among N0 patients, 6 (13.0%), 32 (69.6%), and 8 (17.4%) had RS 0–10, RS 11–25, and RS 26–100, respectively, vs 240 (17.9%), 836 (62.4%), and 264 (19.7%) respectively, in the general 21-gene-tested BC cohort (*p* = 0.84). Similarly, among the N1mi/N1 patients in the study cohort, 1 (7.1%), 12 (85.7%), and 1 (7.1%) had RS 0–10, RS 11–25, and RS 26–100, respectively, vs 115 (16.3%), 457 (64.9%), and 132 (18.8%) respectively, in the general 21-gene-tested BC cohort (*p* = 0.27).

### First vs second BC: clinicopathologic characteristics and RS results

The clinicopathologic characteristics of the first and second BC in the study cohort are presented in Table 2. Except for the significantly older age at testing in the second BC, which reflects the median difference of approximately 5 years between the first and second BC, no other differences in clinicopathologic characteristics were observed (Table 2, Supplementary Table 1).

**Table 2:** Patient and tumor characteristics at the time of first RS and second RS testing in the study cohort.

	First BC *n* = 60	Second BC *n* = 60	p*
Median (IQR) age at testing, years	55.5 (44–62)	60.5 (50–67)	**<0.001**
Median (IQR) tumor size, cm	1.6 (1.1–2.2)	1.4 (1.0–1.8)	0.54
Tumor size category, *n* (%)			0.096^a^
≤1 cm	15 (25.0%)	16 (26.7%)	
>1-2 cm	27 (45.0%)	32 (53.3%)	
>2 cm	18 (30.0%)	9 (15.0%)	
Not available	0 (0%)	3 (5.0)	
Tumor grade category, *n* (%)			0.79^b^
Grade 1	9 (15.0%)	6 (10.0%)	
Grade 2	32 (53.3%)	36 (60.0%)	
Grade 3	10 (16.7%)	9 (15.0%)	
Not available	9 (15.0%)	9 (15.0%)	
Histology, *n* (%)			0.75^c^
IDC	50 (83.3%)	48 (80.0%)	
ILC	5 (8.3%)	10 (16.7%)	
Mucinous/colloid/papillary	2 (3.3%)	1 (1.7%)	
Other/not available	3 (5.0%)	1 (1.7%)	
Nodal status, *n* (%)			0.21^d^
N0	46 (76.7%)	49 (81.7%)	
N1mi	8 (13.3%)	1 (1.7%)	
N1	6 (10.0%)	7 (11.7%)	
Not available	0 (0.0%)	3 (5.0%)	

*BC* breast cancer, *IDC* invasive ductal carcinoma, *ILC* invasive lobular carcinoma, *IQR* interquartile range.

*p value for Wilcoxon signed-rank test and the McNemar’s test for continuous and categorical variables, respectively. Bolded *p* values are statistically significant. McNemar’s test contingency tables are presented in Supplementary Table 1.

^a^For comparing tumor size ≥2 cm to <2 cm.

^b^For comparing grade 1/2 to grade 3.

^c^For comparing IDC to non-IDC histologies.

^d^For comparing N0 to N1/N1mi patients.

**Fig. 2:**
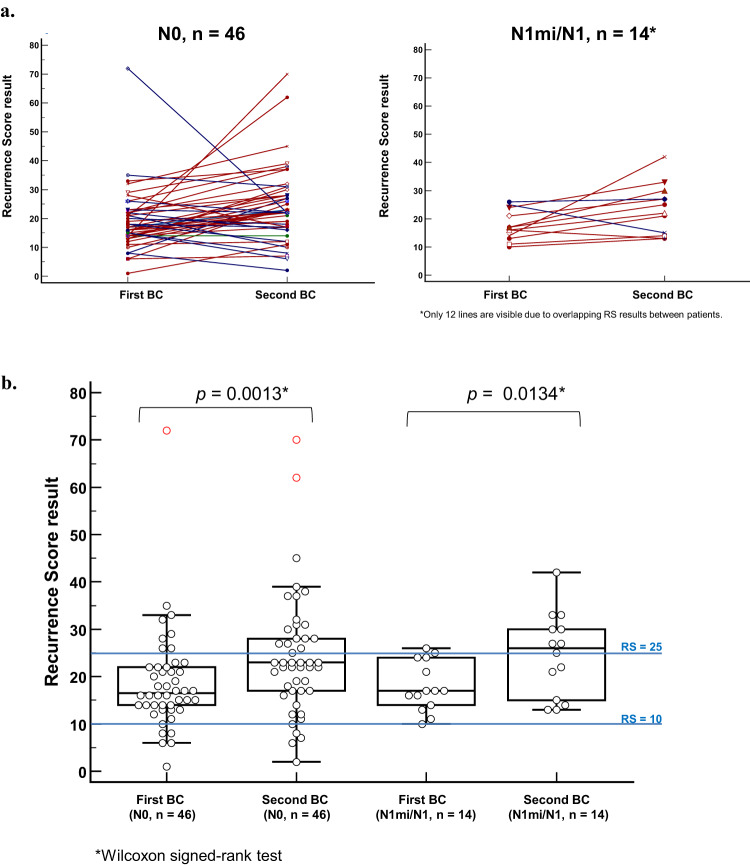
RS results in the first and second BC. **a** RS results in the second versus the first BC for each patient in the study cohort by nodal status. Red markers/lines represent cases where the second RS was higher than the first, blue markers/lines represent cases where the second RS was lower than the first, and green markers/line represent a case with identical RS results in both tests. **b** A Box-and-Whiskers plot showing the RS distribution in the first vs the second BC by nodal status. The horizontal line represents the median, and the box is drawn from the 1^st^ to the 3^rd^ quartile. The upper whisker represents the highest value below the 3^rd^ quartile plus 1.5 × IQR. The lower whisker represents the lowest value above the 1^st^ quartile minus 1.5 × IQR. Red circles represent datapoints that are beyond the 3^rd^ quartile plus 3 × IQR. BC breast cancer.

The differences in the RS results between the first and second RS varied considerably between the patients (Fig. 2a). Overall, in most patients (44 patients, 73.3%), the second RS result was higher than the first with an RS difference of 1–56 units. In a quarter of the patients (15 patients, 25.0%), the second RS was lower than the first, with an RS difference of 1–50 units. One patient (1.7%) had a second RS result that was the same as her first. The median (IQR) RS result for the entire cohort in the second testing was higher at 23 (17–30) vs 17 (14–22) in the first (*p* < 0.001), and correspondingly, in the second testing, more patients (*n* = 24, 40%) were in the RS 26–100 category compared to the first testing (*n* = 9, 15.0%, *p* = 0.0015).

Comparing the first and second RS results in the study cohort by the nodal status (as determined at the first BC) revealed similar results. Among N0 patients, the medians (IQR) were 16.5 (14–22) vs 23 (17–28) in the first and second 21-gene-testing, respectively, *p* = 0.00134; among N1mi/N1 patients, the medians (IQR) were 17 (14–24) vs 26 (15–30), respectively, *p* = 0.013 (Fig. 2b).

### Study cohort: surgeries and treatments

Information regarding the definitive surgery was available for 45 patients (lumpectomy, 41; mastectomy, 4). Of the 46 N0 patients, 28 (61%) underwent lumpectomy and 4 (9%) mastectomy (for the remaining 14 [30%] patients, information was not available). Of the 14 N1mi/N1 patients, 13 (93%) underwent lumpectomy (for 1 [7%] patient, surgery information was not available). Radiation information post-surgery was available for 58 patients. Of the 58 patients, 46 (79%) received radiation therapy. Only one case of post-mastectomy radiation was reported. Information on systemic treatments received after each testing was available for 58 patients in the study cohort and is presented in Table 3 overall and by nodal status. Most patients received ET (80% after the first RS, 87% after the second RS). CT use (with or without ET) was reported for 15 and 37% of patients after the first and second RS testing, respectively (Table 3).

**Table 3:** Systemic treatments received by patients in the study cohort after the first and second 21-gene testing (based on the nodal status as determined in the first BC).

Treatments received, *n* (%)	First BC *n* = 60			Second BC *n* = 60		
	Node-negative *n* = 46	Node-positive *n* = 14	All first BC *n* = 60	Node-negative *n* = 46	Node-positive *n* = 14	All second BC *n* = 60
ET alone	31 (67.4%)	9 (64.3%)	40 (66.7%)	24 (52.2%)	8 (57.1%)	32 (53.3%)
ET plus CT	5 (10.9%)	3 (21.4%)	8 (13.3%)	16 (34.8%)	4 (28.6%)	20 (33.3%)
CT alone	0 (0)	1 (7.1%)	1 (1.7%)	1 (2.2%)	1 (7.1%)	2 (3.3%)
No systemic treatment	8 (17.4%)	1 (7.1%)	9 (15.0%)	2 (4.3%)	1 (7.1%)	3 (5.0%)
Not available	2 (4.3%)	0 (0)	2 (3.3%)	3 (6.5%)	0 (0)	3 (5.0%)

*BC* breast cancer, *CT* chemotherapy, *ET* endocrine therapy.

### Associations between disease characteristics in the first BC, or treatments received thereafter with the second RS results

Contingency tables were used to explore the association between a higher RS in the second vs the first BC and clinicopathologic characteristics of the first BC diagnosis or surgery/treatments received thereafter. The clinicopathologic variables examined were second BC location (ipsilateral vs contralateral), time between the first and second RS (≤ 5, >5 years), age at first testing (≤50, >50 years), tumor size in the first BC (≤2, >2 cm), grade of the first BC (1–2 vs 3 or 1 vs 2–3), nodal status in the first 21-gene testing, and the RS category in the first testing (0–25, 26–100). Variables related to treatment included surgery type (lumpectomy vs mastectomy), whether the patient received radiation therapy (RT) after the first RS result, whether the breast that developed the second BC was irradiated after the first testing, whether the patient received ET after the first RS, whether the patient received ET within 2 years of the second BC, and whether the patient received CT after the first BC. None of the examined associations was found to be statistically significant (Supplementary Table 2).

Notably, the two associations involving RT trended towards statistical significance. RT information was available for 58 patients (46 RT-treated, 12 RT-untreated). Of the 46 RT-treated patients, 36 (78.3%) had second RS that was higher than the first, whereas in 10 patients (21.7%), it was the same/lower. Of the 12 RT-untreated patients, 6 (50%) had a second RS that was higher than the first and the other 6 (50%) did not (*p* = 0.053). Information on whether the second BC occurred in an irradiated breast (i.e., cases where the second BC was ipsilateral and the patient received RT vs all RT-untreated patients plus all contralateral cases) was available for 59 patients (33 with BC in an irradiated breast, 26 with BC in a non-irradiated breast). In 27 of the 33 patients (81.8%) with second BC in an irradiated breast, the RS of the second was higher than the first, and in 6 patients (18.2%) it was same/lower, whereas in the 26 patients with a second BC in a non-irradiated breast 16 (61.5%) had second RS that was higher than the first, and 10 (38.5%) had second RS that was the same/lower (*p* = 0.085) (Supplementary Table 1).

### Clinical outcomes

Of the 60 patients in the study cohort, 56 had follow-up data and were included in the clinical outcome assessment. With a median (IQR) follow-up of 3.5 (2–5) years from the second 21-gene testing, five distant recurrences were reported in the study cohort, of whom none occurred in patients whose second RS was in the 0–11 range. Two and three distant recurrences occurred in patients whose second RS was in the 11–25 and 26–100 range, respectively. The characteristics of these cases are depicted in Table 4. The patients were all <60 years at the first RS testing, and in all, the second RS was higher than the first. Four underwent lumpectomy (for the 5^th^, surgery type was unavailable). Also, Four of the five patients received ET alone after the first treatment, of whom three experienced second BC during their ET (for the 4th patient, this information was not available). Three patients received CT (with or without ET) following the diagnosis of the second BC (Table 4).

**Table 4:** Characteristics of distant recurrence cases in the study cohort.

#	Age at 1^st^ testing, years	Nodal status at 1^st^ testing	RS, 1^st^ testing	Surgery	Treatment received after first testing	Duration between ET discontinuation and 2^nd^ testing	Duration between 1^st^ and 2^nd^ testing, years	RS, 2^nd^ testing	Ipsilateral/ contralateral BC	Treatment received after 2^nd^ testing	Duration between 2^nd^ testing and metastatic disease, years
1	36	N1	10	Lumpectomy	RT + CT	NA	3.5	13	Ipsilateral	ET	2.3
2	56	N0	19	Lumpectomy	RT + ET	Progressed on ET	1.03	22	Ipsilateral	ET	5.0
3	55	N0	21	NA	RT + ET	Progressed on ET	3.9	62	Ipsilateral	CT	0.7
4	47	N1mi	24	Lumpectomy	RT + ET	Progressed on ET	3.2	33	Ipsilateral	ET + CT	1.8
5	59	N0	26	Lumpectomy	RT + ET	NA	5.5	38	Ipsilateral	ET + CT	3.9

*BC* breast cancer, *CT* chemotherapy, *ET* endocrine therapy, *NA* not available, *RS* Recurrence Score, *RT* radiation therapy.

**Fig. 3:**
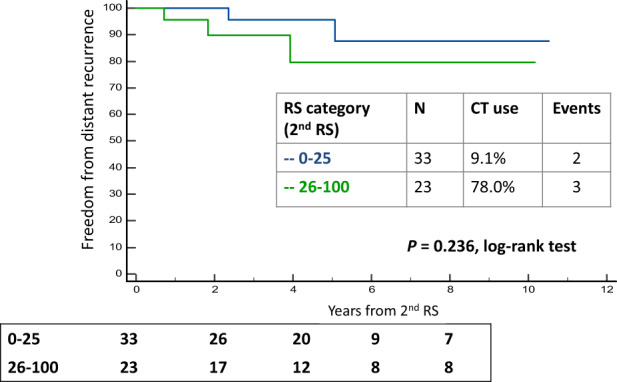
KM curves of freedom from distant recurrence for the study cohort by RS category in the second RS result. The box under the graph presents the number of patients at risk at each time point. One-degree of freedom log-rank *p* values were calculated from all the data. BC breast cancer, CT chemotherapy, RS Recurrence Score.

Kaplan Meier (KM) analysis of freedom from distant recurrence after the second RS for the 56 cases by the RS category (0–25, 26–100) of the second RS demonstrated some separation of the KM curves with seemingly better outcomes in RS 0–25 patients, even though the CT use in RS 0–25 patients was statistically significantly lower than that in the RS 26–100 patients (3/33 [9.1%] vs 18/23 [78.0%], *p* < 0.001). The log-rank test for comparing the KM curves was nonsignificant (*p* = 0.236) (Fig. 3). Similarly, KM analysis of freedom from distant recurrence for these 56 cases according to the relationship between the first and second RS (40 patients whose second RS increased in the second testing vs 16 patients whose second RS was stable/decreased compared to the first testing) also demonstrated some separation of the KM curves with seemingly better outcomes in those whose RS was stable/decreased (the log-rank test was nonsignificant, *p* = 0.152) (Supplementary Fig. 1). Notably, the group with the better outcomes was characterized by overall lower RS results in the second testing compared to the group with poorer outcomes (median [IQR] RS results of 15 [11.3–19.4] vs 27 [22–32.5], respectively, *p* < 0.001).

## Discussion

This is the first report evaluating the RS results in the first and second BC (local recurrence/second primary) in patients with early stage ER + HER2+ disease. The study showed that at their first BC diagnosis, patients who eventually developed second BC had patient/tumor characteristics that were similar to the general 21-gene-tested BC patient population, except for a younger age at diagnosis. The second BC in the study cohort was characterized by higher RS results, but no other significant differences were observed. The study also alluded to a potential prognostic utility of the RS after a second BC.

The current study focused on cases where the first and second BC were both eligible for RS testing (i.e., N0/N1mi/N1, ER+, HER2‒ disease). This patient population was not specifically examined previously. Notably, epidemiologic studies investigating second primary BC (all subtypes) in the US and Canada found that younger age at the initial diagnosis was associated with an elevated risk of a second primary BC^13,14^.

The observed higher RS results in the second BC vs the first suggests that the second BC is more aggressive. Having higher RS results in the second BC was not associated with clinicopathologic characteristics of the first BC or treatments received thereafter, however, the small sample size may have limited the ability to identify statistically significant associations, if such existed. Specifically, ET after the first BC was expected to be associated with an increased RS result in the second BC (i.e., due to the development of ET-resistant tumors), however, no such association was observed. Interestingly, a trend was observed between higher RS result in the second BC and receiving RT as well as having the second BC in an irradiated breast. Several epidemiologic studies demonstrated an association between RT after first BC and having a second primary solid tumor, including second primary BC^15–17^. For example, a recent analysis of the Surveillance, Epidemiology, and End Results (SEER) database involving 13,407 BC patients found an adjusted hazard ratio of 1.268 (95% confidence intervals, 1.112–1.445) for developing second primary contralateral BC in patients who underwent RT vs those who did not^15^. However, data on the association between prior RT, the characteristics of the second BC (including RS result), and clinical outcomes are lacking.

The KM analysis of the study cohort by RS category alludes to a prognostic utility of the RS in this setting, as the KM curves separated. The study sample was too small and the number of patients receiving CT was too low to allow any insights into the role of the RS result as a predictor of CT benefit for patients with second BC. The small sample size probably stems from selection bias with respect to utilizing the test, as second primary BC (for which the test is indicated) is relatively common in HR + BC patients (absolute risk of 13 cases per 10,000 person years with similar risks for developing HR+ and HR− second primary BC^13^). Two main reasons may cause this selection bias. First, by the time patients are diagnosed with a second BC, they are likely to be older and not considered for CT regardless of their RS result. Second, clinicians may be hesitant to offer CT for second BC, based on extrapolation from the CALOR study, where 104 ER+ patients with excised isolated locoregional recurrence were randomized to CT vs no CT, and the evidence did not support CT use in this population^18^. Clearly, the role of CT in second BC, and the role of the RS result as a prognosticator and a predictor of CT benefit in this setting remain clinically important, yet, unresolved. As more BC patients live longer (due to better treatments and because the BC incidence in younger women increases^19,20^), the questions regarding the role of CT and the RS result in the second BC setting are likely to become increasingly clinically relevant. Thus, prospective, well-powered studies for addressing these questions are warranted.

The current study reflects real-life clinical practice, as no exclusion criteria concerning age, tumor characteristics, comorbidities, and treatments were applied. However, the study is limited by its retrospective, nonrandomized design, the small sample size, the small number of events, and a selection bias with respect to the patients being sent to a second 21-gene testing. Moreover, the study considered all cases involved as ‘second BC’ and not second primary BC as even though 21-gene testing is not indicated for local recurrence, such recurrences (for ipsilateral cases) could not be ruled out with the available data. The study is also limited by the lack of genetic information data (e.g., information on whether the patients have germline pathogenic variants in *BRCA1/2*) as such information could have impacted treatment decisions and outcomes. Lastly, during the study period (2006–2020), the RS threshold for recommending chemotherapy changed following the publication of the findings from the TAILORx and RxPONDER studies, which could have impacted treatment decisions depending on the timing of the first and second BC^1,2,6^.

In conclusion, this exploratory retrospective analysis of the CHS registry suggests that second BC is characterized by overall higher RS results compared to the first BC, with no other significant differences in clinicopathologic characteristics, and implies that the RS may have a potential prognostic role for the RS result in this setting. Additional studies are warranted.

## Methods

### Study design, patient population, and data sources

This retrospective exploratory analysis of the prospectively-designed CHS registry, included a BC cohort (the ‘study cohort’), consisting of all ER + HER2‒ BC patients who underwent 21-gene testing through CHS between 1/2006 and 12/2020, had N0, N1mi, or N1 disease at the time of their first testing, and for whom at least 2 RS results were identified, which were performed more than a year apart. No exclusion criteria were applied.

The study cohort was compared to a control cohort extracted from CHS registry-derived previously-reported cohorts for which treatment and clinical outcome data were available^9–11^. This control cohort represented the general 21-gene-tested BC patient population (the ‘general 21-gene-tested BC cohort’). Specifically, the control cohort was generated using 2 previously reported cohorts: A N0 cohort which included patients tested between 2006 and 2009 (*n* = 1365)^11^, and a N1mi/N1 cohort which included patients tested between 2006 and 2011 (*n* = 709)^9^. Male patients and those included in the current study cohort were excluded yielding a final ‘general 21-gene-tested BC cohort’ consisting of 1240 N0 and 704 N1mi/N1 patients.

Data sources used for the analysis included the Oncotest database (for RS results and patient/tumor characteristics) and medical records for treatments received, recurrence, and death.

The study was approved by the institutional review board (IRB) of the CHS Community Division and the participating medical centers and was conducted in accordance with the declaration of Helsinki. The study was granted a waiver for obtaining patient consent due to its retrospective design.

### Statistical analysis

Descriptive statistics were used to summarize clinicopathologic characteristics and adjuvant treatment decisions. Mann-Whitney and chi-square tests were used to compare continuous and categorical variables, respectively, between the cohorts. Wilcoxon signed-rank test and the McNemar’s test were used to compare continuous and categorical variables, respectively between the first and second BC in the study cohort. The association between clinicopathologic characteristics/treatment and increase in RS result from the first to the second testing was analyzed using contingency tables and chi-square/Fisher’s exact test. KM survival curves for freedom from distant recurrence were compared using the log-rank test. Freedom from distant recurrence was determined from the second 21-gene test. Patients were censored at the time of last follow-up, date of medical record review, or time of death (due to any cause).

Data calculation and statistical analysis were performed using R (https://www.r-project.org/, version 4.2.1^21^) or MedCalc^®^ version 20.111 (MedCalc Software Ltd., Ostend Belgium). *p* ≤ 0.05 was considered statistically significant and all tests were two-sided.

### Reporting summary

Further information on research design is available in the Nature Research Reporting Summary linked to this article.

### Supplementary information

Supplementary tables and figure

Reporting Summary

## Data Availability

The datasets generated during and/or analyzed during the current study are available from the corresponding author on reasonable request.
