# Supplementary material for: Second breast cancer: recurrence score results, clinicopathologic characteristics, adjuvant treatments, and outcomes—exploratory analysis of the Clalit registry
Source: NPJ Breast Cancer. 2023 Sep 30;9:79. doi: 10.1038/s41523-023-00586-3 (PMC10541873; doi:10.1038/s41523-023-00586-3)
Supplement: Supplementary file 1 — Supplementary tables and figure [file 41523_2023_586_MOESM1_ESM.pdf]

Supplementary Materials

Supplementary Table 1. Contingency table analysis to evaluate patient and tumor characteristics at the time of first and second RS testing in the study cohort.

Parameter			Second BC			*p value
**Tumor size[1]**			**≤2 cm**	**> 2 cm**	**Total**	
	First BC	**≤2 cm**	35	5	**40**	0.096
		**> 2 cm**	13	4	**17**	
		**Total**	**48**	**9**	**57**	
**Tumor grade[2]**			**Grade 1/2**	**Grade 3**	**Total**	
	First BC	**Grade 1/2**	30	6	**36**	0.79
		**Grade 3**	8	2	**10**	
		**Total**	**38**	**8**	**46**	
**Histology**			**IDC**	**Not IDC**	**Total**	
	First BC	**IDC**	44	6	**50**	0.75
		**Not IDC**	4	6	**10**	
		**Total**	**48**	**12**	**60**	
**Nodal status[3]**			**N0**	**N1mi/N1**	**Total**	
	First BC	**N0**	38	5	**43**	0.21
		**N1mi/N1**	11	3	**14**	
		**Total**	**49**	**8**	**57**	
**Recurrence**			**0-25**	**26-100**	**Total**	
**Score category**	First BC	**0-25**	33	18	**51**	0.0015
		**26-100**	3	6	**9**	
		**Total**	**36**	**24**	**60**	

BC breast cancer, *IDC* invasive ductal carcinoma.

**p* value was calculated using the McNemar's test.

[1] Tumor size information in either or both BC instances was not available for 3 patients.

[2] Grade information in either or both BC instances was not available for 14 patients.

[3] Nodal status information in either or both BC instances was not available for 3 patients

Supplementary Table 2. Contingency table analysis to evaluate the association between clinical characteristics or adjuvant treatments and having a second RS result that is higher than the first.

Parameter	Increase in the second RS result, *n*	No increase in the second RS result, *n* [1]	Total, *n*	**p* value
**Second BC location**				
**Ipsilateral**	32	9	**41**	
**Contralateral**	12	7	**19**	0.23
**Total**	**44**	**16**	**60**	
**Time between the first and second 21-gene testing**				
**≤5 years**	22	6	**28**	
**<5 years**	22	10	**32**	0.40
**Total**	**44**	**16**	**60**	
**Age at the first 21-gene testing**				
**≤50 years**	16	6	**32**	
**<50 years**	28	10	**38**	0.94
**Total**	**44**	**16**	**60**	
**Tumor size at the first 21-gene testing**				
**≤2 cm**	28	14	**42**	
**<2 cm**	16	2	**18**	0.11
**Total**	**44**	**16**	**60**	
**Grade at the first 21-gene testing**				
**1-2**	27	14	**41**	
**3**	9	1	**10**	0.25
**Total**	**36**	**15**	**51**	
**Grade at the first 21-gene testing**				
**1**	6	3	**9**	
**2-3**	30	12	**42**	1.0
**Total**	**36**	**15**	**51**	
**Nodal status at the first 21-gene testing**				
**Node negative**	32	14	**46**	
**Node positive**	12	2	**14**	0.31
**Total**	**44**	**16**	**60**	
**RS category of the first RS result**				
**0-25**	39	12	**51**	
**26-100**	5	4	**9**	0.23
**Total**	**44**	**16**	**60**	
**Surgery type**				
**Lumpectomy**	31	10	**41**	
**Mastectomy**	2	2	**4**	0.29
**Total**	**33**	**12**	**45**	
**Patient received RT after the first RS result**				
**Yes**	36	10	**46**	
**No**	6	6	**12**	0.053
**Total**	**42**	**16**	**58**	
**Patient received RT after the first RS result, to the breast where the second primary BC was eventually identified**				
**Yes**	27	6	**33**	
**No**	16	10	**26**	0.085
**Total**	**43**	**16**	**59**	

**Patient received ET after the first RS result**				
**Yes**	35	13	**48**	
**No**	7	3	**10**	1.0
**Total**	**42**	**16**	**58**	
**Patient received ET within 2 years of the first RS result**				
**Yes**	21	6	**27**	
**No**	11	5	**16**	0.52
**Total**	**32**	**11**	**43**	
**Patient received CT after the first RS result**				
**Yes**	5	4	**9**	
**No**	37	12	**49**	0.24
**Total**	**42**	**16**	**58**	

CT chemotherapy, *ET* endocrine therapy, *RS Recurrence Score, RT* radiation therapy.

**p* value was calculated using chi-square test or Fisher's exact test (when at least one subgroup included <5 patients).

[1]In one case, the second RS was the same as the first, and in all others, the second RS was lower than the first.

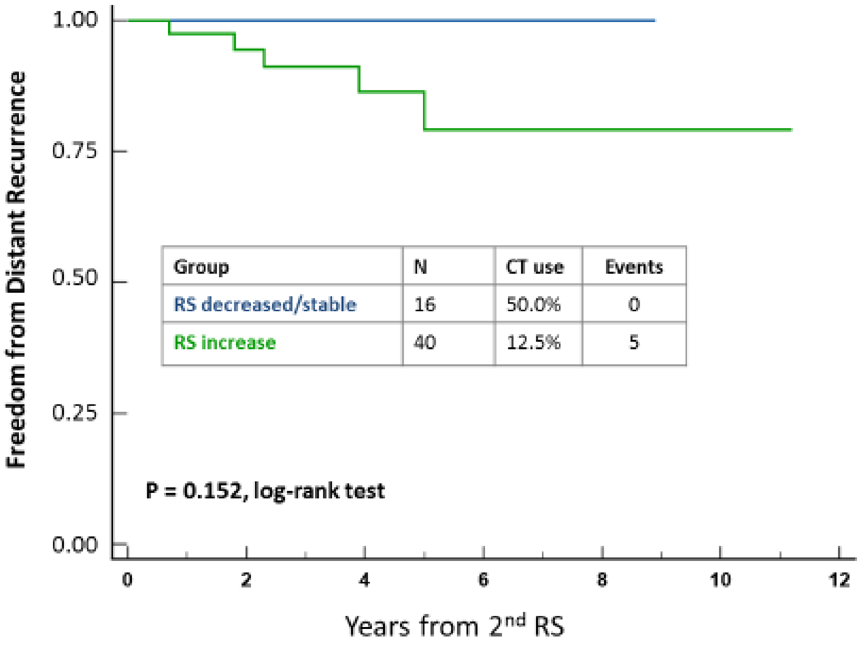

RS decrease	16	10	9	4	1
RS increase	40	29	18	8	3

Supplementary Figure 1. KM curves of freedom from distant recurrence for the study cohort by the relationship between the first and second RS (increased vs decreased/stable RS). The box under the graph presents the number of patients at risk at each time point. One-degree of freedom log-rank *p* values were calculated from all the data. *BC* breast cancer, *CT* chemotherapy, *RS* Recurrence Score.
