# Supplementary material for: Second breast cancer: recurrence score results, clinicopathologic characteristics, adjuvant treatments, and outcomes—exploratory analysis of the Clalit registry
Source: NPJ Breast Cancer. 2023 Sep 30;9:79. doi: 10.1038/s41523-023-00586-3 (PMC10541873; doi:10.1038/s41523-023-00586-3)
Supplement: Supplementary file 2 — Reporting Summary [file 41523_2023_586_MOESM2_ESM.pdf]

nature portfolio

Corresponding author(s): Shlomit S. Shachar

Last updated by author(s): 2023-5-30

Reporting Summary

Nature Portfolio wishes to improve the reproducibility of the work that we publish. This form provides structure for consistency and transparency in reporting. For further information on Nature Portfolio policies, see our Editorial Policies and the Editorial Policy Checklist.

Statistics

For all statistical analyses, confirm that the following items are present in the figure legend, table legend, main text, or Methods section.

n/a	Confirmed	
☐	☒	The exact sample size (*n*) for each experimental group/condition, given as a discrete number and unit of measurement
☐	☒	A statement on whether measurements were taken from distinct samples or whether the same sample was measured repeatedly
☐	☒	The statistical test(s) used AND whether they are one- or two-sided<br>*Only common tests should be described solely by name; describe more complex techniques in the Methods section.*
☐	☒	A description of all covariates tested
☐	☒	A description of any assumptions or corrections, such as tests of normality and adjustment for multiple comparisons
☐	☒	A full description of the statistical parameters including central tendency (e.g. means) or other basic estimates (e.g. regression coefficient) AND variation (e.g. standard deviation) or associated estimates of uncertainty (e.g. confidence intervals)
☐	☒	For null hypothesis testing, the test statistic (e.g. F, t, r) with confidence intervals, effect sizes, degrees of freedom and P value noted<br>*Give P values as exact values whenever suitable.*
☒	☐	For Bayesian analysis, information on the choice of priors and Markov chain Monte Carlo settings
☒	☐	For hierarchical and complex designs, identification of the appropriate level for tests and full reporting of outcomes
☒	☐	Estimates of effect sizes (e.g. Cohen's d, Pearson's r), indicating how they were calculated

Our web collection on statistics for biologists contains articles on many of the points above.

Software and code

Policy information about availability of computer code

Data collection	Not relevant.
Data analysis	Softwares used for the statistical analyses: R (https://www.r-project.org/, version 4.2.121) and MedCalc® version 20.111 (MedCalc Software Ltd., Ostend Belgium).

For manuscripts utilizing custom algorithms or software that are central to the research but not yet described in published literature, software must be made available to editors and reviewers. We strongly encourage code deposition in a community repository (e.g. GitHub). See the Nature Portfolio guidelines for submitting code & software for further information.

Data

Policy information about availability of data

All manuscripts must include a data availability statement. This statement should provide the following information, where applicable:

- Accession codes, unique identifiers, or web links for publicly available datasets
- A description of any restrictions on data availability
- For clinical datasets or third party data, please ensure that the statement adheres to our policy

The datasets generated during and/or analyzed during the current study are available from the corresponding author on reasonable request.

Research involving human participants, their data, or biological material

Policy information about studies with human participants or human data. See also policy information about sex, gender (identity/presentation), and sexual orientation and race, ethnicity and racism.

Reporting on sex and gender	The study cohort included only females who were also women (no trans women or trans men). Therefore the reporting by sex and gender is the same in this case.
Reporting on race, ethnicity, or other socially relevant groupings	The study was a multicenter Israeli study. Ethnicity and race data were not available.
Population characteristics	The study cohort included 60 patients whose relevant characteristics are presented in Tables 1 and 2.
Recruitment	This was a retrospective study. The study included all patients in the CHS registry who met the eligibility criteria.
Ethics oversight	The study was approved by the institutional review board (IRB) of the CHS Community Division and the participating medical centers and was conducted in accordance with the declaration of Helsinki. The study was granted a waiver for obtaining patient consent due to its retrospective design.

Note that full information on the approval of the study protocol must also be provided in the manuscript.

Field-specific reporting

Please select the one below that is the best fit for your research. If you are not sure, read the appropriate sections before making your selection.

☒ Life sciences ☐ Behavioural & social sciences ☐ Ecological, evolutionary & environmental sciences

For a reference copy of the document with all sections, see nature.com/documents/nr-reporting-summary-flat.pdf

Life sciences study design

All studies must disclose on these points even when the disclosure is negative.

Sample size	The study included 60 patients.
Data exclusions	For the study cohort, all patients who met the inclusion criteria were included. No exclusion criteria were applied.
Replication	Not relevant. Real-world data.
Randomization	Not relevant. Real-world data.
Blinding	Not relevant. Real-world data.

Reporting for specific materials, systems and methods

We require information from authors about some types of materials, experimental systems and methods used in many studies. Here, indicate whether each material, system or method listed is relevant to your study. If you are not sure if a list item applies to your research, read the appropriate section before selecting a response.

Materials & experimental systems

n/a	Involved in the study	
☒	☐	Antibodies
☒	☐	Eukaryotic cell lines
☒	☐	Palaeontology and archaeology
☒	☐	Animals and other organisms
☒	☐	Clinical data
☒	☐	Dual use research of concern
☒	☐	Plants

Methods

n/a	Involved in the study	
☒	☐	ChIP-seq
☒	☐	Flow cytometry
☒	☐	MRI-based neuroimaging
